# Eukaryotic translation initiation factor 3 subunit D overexpression is associated with the occurrence and development of ovarian cancer

**DOI:** 10.1002/2211-5463.12137

**Published:** 2016-10-31

**Authors:** Yaying Lin, Rongrong Zhang, Ping Zhang

**Affiliations:** ^1^ Department of GynecologyXinhua HospitalShanghai Jiao Tong University School of MedicineChina

**Keywords:** cell cycle, EIF3D, ovarian cancer, proliferation

## Abstract

Ovarian cancer is the most common cause of gynaecological cancer‐associated death; thus, promising biomarkers and new therapeutic targets for ovarian cancer must be explored. Here, we report that eukaryotic translation initiation factor 3 subunit D (EIF3D), a member of the EIF3 family, was overexpressed in ovarian cancer clinical tissues. Furthermore, the expression of EIF3D was correlated with the International Federation of Gynecology and Obstetrics stage and pathological differentiation stage. 3‐(4,5‐dimethylthylthiazol‐2‐yl)‐2 (MTT) and colony formation assays revealed that the lentivirus‐mediated knockdown of EIF3D suppresses cell proliferation in the ovarian tumour cell lines CAOV‐3 and SKOV‐3. Flow cytometry revealed that cells were arrested at the G2/M phase of the cell cycle and that cyclin‐dependent kinase 1 was also altered after EIF3D silencing. The results presented here demonstrate that EIF3D may play an important role in the occurrence and development of ovarian cancer.

**Abbreviations**
BRAF: B‐Raf proto‐oncogene, serine/threonine kinase
BRCA1/2: BRCA1/2, DNA repair associated
CDK12: cyclin‐dependent kinase 12
CDK1: cyclin‐dependent kinase 1
EIF3D: eukaryotic translation initiation factor 3 subunit D
FIGO: The International Federation of Gynecology and Obstetrics
KRAS: KRAS proto‐oncogene, GTPase
Lv‐shRNA: lentivirus‐mediated shRNA
MTT: 3‐(4,5‐dimethylthylthiazol‐2‐yl)‐2
NF1: neurofibromin 1
PCI/MPN: PCI/MPN domain protein complexes
PTEN: phosphatase and tensin homologue
shRNA: short hairpin RNA
TP53: tumour protein p53
VEGF: vascular endothelial growth factor

Ovarian cancer is the most common cause of death associated with gynaecologic malignancies, and 225 500 women are newly diagnosed worldwide annually 1. Because of its late presentation, ovarian cancer is frequently metastatic throughout the abdomen upon initial diagnosis. The current standard treatment for ovarian cancer is optimal surgical resection followed by platinum‐based chemotherapy 2. Although a considerable proportion of women attain complete response after periodic chemotherapy, most experience chemo‐resistance and relapse after 28 months 3. Ovarian cancer arises and develops through a multistep process in which genetic and epigenetic aberrations occur and accumulate and subsequently lead to malignant transformation. TP53, BRCA1/2, NF1, CDK12, PTEN, BRAF, KRAS and VEGF expression is aberrant in ovarian cancer, and many of these proteins are used as biomarkers or therapeutic targets 2, 4, 5, 6. However, the mechanism underlying the development of ovarian cancer is still unknown, and sensitive and specific biomarkers are not available.

Protein synthesis regulation at translation initiation is a common and crucial method by which eukaryotes control cell proliferation and differentiation, and dysregulation of this process may contribute to cell immortalization. In this step, eukaryotic translation initiation factors (EIFs) play important roles in mediating mRNA binding to the 40S ribosomal subunit 7. Many EIFs appear to be overexpressed or underexpressed in various tumours 8, 9, 10, meaning that EIF deregulation is associated with tumourigenesis via the dysregulation of the signal transduction pathways that regulate protein synthesis or the alteration of EIF pathways. EIF3 is the largest and most complex initiation factor and includes 13 nonidentical subunits designated EIF3a to EIF3m 11. The main function of EIF3 is to orchestrate the formation of 43S–48S preinitiation complexes and act as a scaffold. A biochemical study suggested that six subunits in mammals (A, B, C, E, F and H) form the ‘core’ subunits and play a leading role in translation 12. Recently, the core of EIF3 was proposed to include the PCI/MPN octamer, which contains subunits A, C, E, F, H, K, L and M as determined by cryo‐EM studies 13, 14. Especially, EIF3A and EIF3C are more important for the assembly of EIF3s and PCIs 15.

The eukaryotic translation initiation factor 3 subunit D (EIF3D), also named EIF3‐P66 or EIF3‐zeta, is a component of EIF3 complexes. Moe1, the homologue of EIF3D in fission yeast, is required for stable associations among EIF3 subunits 16. EIF3D inhibits HIV replication, suggesting an antiviral function 17. Recent studies have examined the relationship between EIF3D and tumours. EIF3D knockdown suppresses the proliferation of human melanoma cells 18 and prostate cancer cells 19, and similar phenomena have been observed in EIF3D‐knockdown HCT116 colon cancer cells 20, indicating EIF3D's potential role as an oncogene. Moreover, EIF3D knockdown may inhibit breast cancer cell proliferation and invasion by suppressing the Wnt/β‐catenin pathway 21. However, the mechanisms by which EIF3D promotes oncogenesis remain unclear, and the effects of EIF3D in ovarian cancer have not been reported.

## Methods

### Cell culture

The human ovarian cancer cell lines CAOV‐3 and SKOV‐3 and human embryonic kidney cells 293T were obtained from the Cell Bank of the Chinese Academy of Science (Shanghai, China). SKOV‐3 cells were cultured in McCoy's 5A medium (M8403; Sigma Aldrich, St. Louis, MO, USA) supplemented with 10% heat‐inactivated FBS(S1810; Biowest, Nuaillé, France) in 5% CO_2_ at 37 °C. The CAOV‐3 cells were cultured in RPMI‐1640 medium (SH30809.01B; Hyclone, Waltham, MA, USA) supplemented with 10% heat‐inactivated FBS (S1810; Biowest) in 5% CO_2_ at 37 °C. The 293T cells were cultured in Dulbecco's modified Eagle's medium (SH30243.01B; Hyclone) supplemented with 10% heat‐inactivated FBS (S1810; Biowest) in 5% CO_2_ at 37 °C.

### EIF3D gene silencing with short hairpin RNA mediated by lentivirus

Two short hairpin RNA (shRNA) target sequences were designed for the human eIF3D gene (NM_003753.3): sequence 1 (EIF3D shRNA S1) (5′‐GACGACATGGATAAGAATGAACTCGAGTTCATTCTTATCCATGTCGTCTTTTTT‐3′) and sequence 2 (EIF3D shRNA S2) (5′‐GCGTCATTGACATCTGCATGACTCGAGTCATGCAGATGTCAATGACGCTTTTTT‐3′). Scramble shRNA (5′‐GCGGAGGGTTTGAAAGAATATCTCGAGATATTCTTTCAAACCCTCCGCTTTTTT‐3′) was used as a control to test the nonspecific effects on gene expression. Plasmid DNA sequencing was performed as a validation step, and then the designed shRNAs were inserted into the lentiviral expression vector pFH‐L (Shanghai Hollybio, Shanghai, China) along with the GFP reporter gene. The modified pFH‐L vector and packing plasmids pVSVG‐I and pCMVΔR8.92 (Shanghai Hollybio) were triple transfected into the 293T cells for 48 h to produce the recombinant lentiviruses using Lipofectamine 2000 (Invitrogen, Carlsbad, CA, USA) according to the manufacturer's instructions. The CAOV‐3 cells and SKOV‐3 cells (50 000 cells per well in six‐well plates) were transfected with the purified recombinant lentiviruses for 96 h at the corresponding MOI (multiplicity of infection; 25 for CAOV‐3 cells and 20 for SKOV‐3 cells), and the infection efficiencies were confirmed by fluorescence microscopy.

### qPCR assay

Total RNA was extracted from the CAOV‐3 and SKOV‐3 cells after 5 days of infection with TRIzol reagent (Gibco, Carlsbad, CA, USA) according to the manufacturer's instructions, and 5 μg extracted RNA was utilized for the cDNA synthesis with SuperScript II RT at 200 units·mL^−1^ (Invitrogen). A qPCR assay was performed using the BioRad Connect Real‐Time PCR platform (BioRad, Hercules, CA, USA) and the following reaction mixture: 10 μL 2× SYBR Premix Ex Taq, 0.8 μL forward and reverse primers (2.5 μm), 5 μL cDNA and 4.2 μL ddH_2_O. The following EIF3D primers were used: 5′‐CTGGAGGAGGGCAAATACCT‐3′ forward and 5′‐CTCGGTGGAAGGACAAACTC‐3′ reverse. The following cyclin‐dependent kinase 1 (CDK1) primers were used: 5′‐CAGACTAGAAAGTGAAGAGGAAGG‐3′ forward and 5′‐ACTGACCAGGAGGGATAGAATC ‐3′ reverse. The following β‐actin primers were used: 5′‐GTGGACATCCGCAAAGAC‐3′ forward and 5′‐AAAGGGTGTAACGCAACTA‐3′ reverse. The qPCR procedure was performed as follows: initial denaturation at 95 °C for 1 min; 40 cycles of denaturation at 95 °C for 5 s; and then an annealing extension at 60 °C for 20 s. The absorbance values were measured at the extension stage. Data were analysed by the $2^{-\Delta \Delta C_{t}}$ method, and the expression level of each mRNA was presented as *C*_T_ values, which indicated the threshold PCR cycle number at which an amplified product was first detected: Δ*C*_T_ = *C*_T_ EIF3D‐*C*_T_ β‐actin.

### Western blot

Five days after inoculation with the lentivirus, total protein extraction was performed on the CAOV‐3 and SKOV‐3 cells using 2× SDS sample buffer [100 mm Tris‐HCl (pH 6.8), 10 mm EDTA, 4% SDS and 10% glycine]. After quantification by (bicinchoninic acid, 30 μg of protein from each extract was loaded and electrophoresed on 10% polyacrylamide SDS gel at 50 V for 3 h and then transferred to polyvinylidene fluoride membranes (Millipore, Billerica, MA, USA) at 300 mA for 1.5 h. The membranes were blocked for 1 h in TBST [Tris‐buffered saline with Tween‐20; 20 mm Tris (pH 7.6), 150 mm NaCl and 0.01% Tween‐20] with 5% skim milk prior to incubation in EIF3D rabbit primary antibody (Abcam, Cambridge, UK; ab155419, dilution 1 : 1000) or GAPDH rabbit primary antibody (Proteintech, Chicago, IL, USA; 10494‐1‐AP, dilution 1 : 40 000) at 4 °C overnight with constant stirring. The membranes were washed in TBST three times on the second day and probed for 2 h at room temperature with HRP (horseradish peroxidase)‐labelled goat anti‐rabbit secondary antibody (Santa Cruz, CA, USA; Sc‐2054, dilution 1 : 5000). The membranes were observed after a chemiluminescence agent was added to determine the relative concentration of the protein.

### MTT assay

The CAOV‐3 or SKOV‐3 cells were seeded in 96‐well plates at a density of 3000 cells per well grouped by Con, Lv‐shCon and Lv‐shEIF3D. 3‐(4,5‐dimethylthylthiazol‐2‐yl)‐2 (MTT) were added into each well at 5 mg·mL^−1^ after the indicated times (1–5 days). Four hours later, the mixtures were incubated with acidic isopropanol (10% SDS, 5% isopropanol and 0.01 m HCl) overnight at 37 °C to terminate the reaction. Data were obtained via spectrophotometry (BioRad) at 595 nm.

### Colony formation assay

The CAOV‐3 cells in the Con, Lv‐shCon and Lv‐shEIF3D groups undergoing logarithmic growth were detached from the culture dishes. After the appropriate dilutions, the cells were plated into six‐well plates at 700 cells per well. After culture for 10 days, the cells were fixed by paraformaldehyde and stained with crystal violet. The cell clusters were calculated and represent the clonality of the cells.

### Flow cytometry

The CAOV‐3 cells infected by recombinant lentiviruses for 3 days were reseeded into the 6‐cm dishes at approximately 200 000 cells per dish. After culturing for 40 h at 37 °C and 5% CO_2_, the cells were digested and harvested at 80% confluence. All samples were washed in 4 °C PBS and then fixed in 70% cold alcohol. Subsequently, the cells were centrifuged and suspended in PI/RNase/PBS (100 μg·mL^−1^ propidium iodide and 10 μg·mL^−1^ RNase A) solution. After staining for 30 min at room temperature, the cell cycle was analysed via flow cytometry.

### Immunohistochemistry

Ovarian adenoma, borderline lesion and cancer tissues were collected from 2009 April to 2011 October at Xinhua Hospital. All paraffin‐embedded samples were sliced into 3‐μm sections and adhered on the slides. After deparaffinization in different density gradients of dimethylbenzene, the slides were rehydrated and antigens were retrieved using citric acid buffer (pH 7.8, 0.1 m) for 24 min at approximately 82 °C. The slides were uniformly covered with endogenous peroxidase blocking solution (Hologic, Beijing, China) for 15 min at room temperature to block the activation of endogenous peroxidase. After incubation in EIF3D primary antibody (ab155419; Abcam; dilution 1 : 200) overnight at 4 °C, the slides were gently washed with nonimmune water, incubated with biotin‐bound secondary antibody for 10 min at room temperature and then incubated with streptavidin–peroxidase for 5 min. All slides were then stained with haematoxylin and washed. An immunohistochemistry assessment was performed by the first author after the slides were dried and cleared. The staining intensity and proportion of positively stained area was assessed using the following criteria. For the staining intensity, a score of 0 indicates negative staining, 1 indicates weak staining, 2 indicates moderate staining and 3 indicates strong staining. Regarding the proportion of the positively stain area, 0 indicates no staining, 1 indicates 0–20% staining, 2 indicates 21–60% staining and 3 indicates 61–100% staining. Ten random fields were assessed for each slide, and the average value was obtained. The terminal score was obtained from the sum of the staining intensity score and the proportion of positively stained area: 0 indicates a negative score, 1 indicates a weak score, 2–4 indicates a moderate score and 5–6 indicates a strong score.

### Statistical analysis

The data from the immunohistochemical analysis for EIF3D were analysed using a Kruskal–Wallis nonparametric one‐way ANOVA with a Nemenyi pairwise multiple test. The results are listed as the mean ± SD of at least three determinations, and statistical comparisons were performed via Student's *t*‐test. *P*‐values < 0.05 were considered significant. Statistical analyses were performed with SAS 8.0 (SAS Institute Inc., Cary, NC, USA).

### Ethics statement

Informed consent was obtained from all patients, and the work described herein was performed in accordance with the Code of Ethics of the World Medical Association (Declaration of Helsinki) for experiments involving humans. The study methodologies received approval from the appropriate local ethics committee (No. XHEC‐C‐2016‐309).

## Results

### Increased EIF3D expression related to the occurrence and development of ovarian cancer

**Figure 1:**
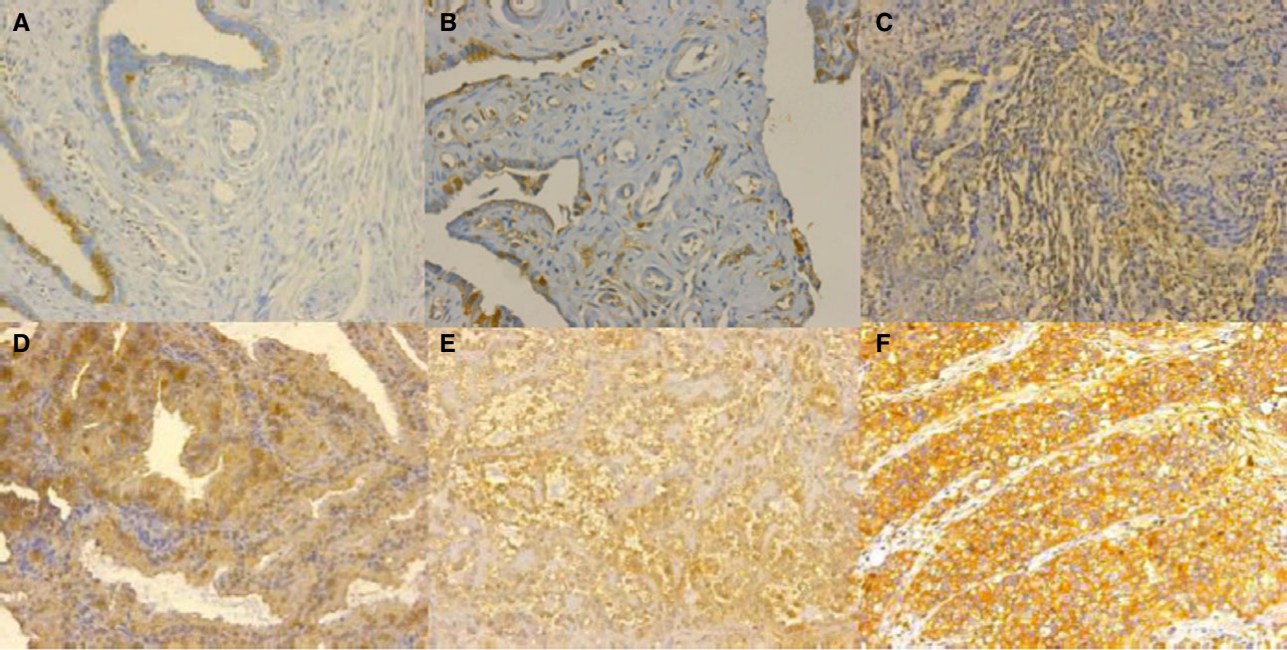
EIF3D expression in tissues. EIF3D is mainly located in cytoplasm, and its expression is increasing consistent with the occurrence and development of ovarian cancer (A–F, 200×). In benign ovarian cystadenoma (A), the stain of EIF3D was extremely weak and located in margin with a few areas. In borderline cystadenoma (B), EIF3D expression began to elevate but still weak compared with the serous cystadenocarcinoma (C), endometrial adenocarcinoma (D) and clear cell carcinoma (E), while reached the highest in colon metastasis focus (F).

The immunohistochemical analysis detected EIF3D expression in the ovarian adenoma, borderline lesion and cancer (serous cystadenocarcinoma, clear cell carcinoma and endometrial adenocarcinoma) tissues. As shown in Fig. 1, EIF3D was mainly located in the cytoplasm. In benign ovarian cystadenoma (Fig. 1A), EIF3D expression was extremely weak or even negative, whereas it began to increase in borderline cystadenoma (Fig. 1B) but remained weak compared with the ovarian cancers (Fig. 1C–F, Table 1, *P* < 0.01). EIF3D staining was strongest in the colon metastases (Fig. 1F).

**Table 1:** Expression of EIF3D in ovarian cancer

Feature	*n*	EIF3D expression				*P*‐value^a^
		Negative 0	Weak 1	Moderate 2–4	Strong 5–6	
Tissue type						
Benign ovarian cystadenoma	42	19	16	6	1	< 0.0001
Borderline cystadenoma	23	2	5	13	3	
Ovarian cancer	81	3	6	32	40	
Subtypes of ovarian cancer						
Serous cystadenocarcinoma	42	2	3	15	22	0.3806
Clear cell carcinoma	18	1	1	10	6	
Endometrial adenocarcinoma	21	0	2	7	12	
FIGO stage						
IA	1	0	0	1	0	0.0172
IB	3	0	1	0	2	
IC	2	1	1	0	0	
IIA	5	0	2	3	0	
IIB	8	1	1	2	4	
IIC	10	0	0	6	4	
IIIA	9	1	0	5	3	
IIIB	9	0	1	2	6	
IIIC	19	0	0	10	9	
IV	15	0	0	3	12	
Tumour differentiation						
G1	19	2	4	7	6	0.0305
G2	26	0	1	13	12	
G3	36	1	1	12	22	

a Analysed by the Kruskal–Wallis nonparametric one‐way ANOVA followed by the Nemenyi pairwise multiple test. *P* < 0.05 was considered significant.

Correlations were not observed between EIF3D expression and ovarian cancer subtypes (Table 1, *P* = 0.3806). However, when grouped by the International Federation of Gynecology and Obstetrics (FIGO) stages, EIF3D expression increased from I to IV (Table 1, *P* = 0.0172). Furthermore, tumour differentiation indicated that EIF3D expression gradually increased from G1 to G3 (Table 1, *P* = 0.0305).

### Lv‐shRNA decreased EIF3D expression in ovarian cancer cells

**Figure 2:**
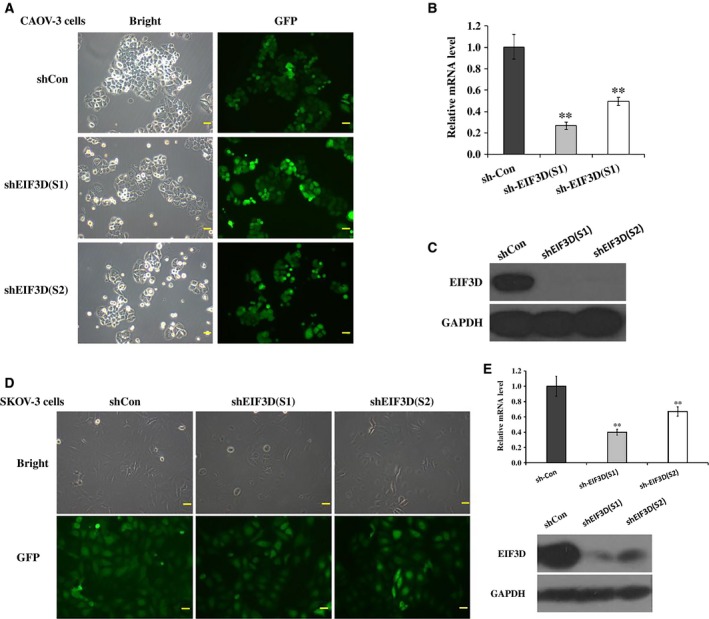
Knockdown EIF3D in CAOV‐3 and SKOV‐3 cells through Lv‐shRNA. CAOV‐3 and SKOV‐3 cells were infected with indicated recombinant lentiviruses for 96 h and their infection efficiency were examined by fluorescence microscopy. Cells observed through bright light and green fluorescence of GFP were shown (A, D). mRNA levels of EIF3D in CAOV‐3 cells were detected by qPCR and β‐actin was used as internal reference (B), as well as in SKOV‐3 (E). EIF3D protein levels were analysed by western blot and GAPDH was used as internal reference (C, E). **P < 0.01, compared with shCon.

To determine the function of EIF3D in ovarian cancer *in vitro*, we knocked down the gene using lentivirus‐mediated RNAi in the ovarian cancer cell lines CAOV‐3 and SKOV‐3. An analysis of the number of cells exhibiting GFP fluorescence indicated that the infection efficiency was greater than 80% (Fig. 2A, D). The knockdown efficiency was evaluated by qPCR and western blot. The EIF3D mRNA levels in the CAOV‐3 cells were significantly reduced under treatment with lentivirus‐mediated shRNA (Lv‐shRNA) sequence 1 (*P* = 0.0051, knockdown efficiency = 73.4%) and sequence 2 (*P* = 0.0065, knockdown efficiency = 50.6%) compared with the Lv‐shCon treatment (Fig. 2B). The EIF3D mRNA levels in the SKOV‐3 cells were reduced under treatment with Lv‐shRNA sequence 1 (*P* = 0.0002, knockdown efficiency = 61.5%) and sequence 2 (*P* = 0.0065, knockdown efficiency = 32.7%) (Fig. 2E), and the EIF3D protein was down‐regulated in CAOV‐3 cells (Fig. 2C) and SKOV‐3 cells (Fig. 2E) according to the western blot assay. In conclusion, EIF3D was knocked down by Lv‐shRNA in ovarian cancer cells.

### Cell proliferation was inhibited after EIF3D gene silencing

**Figure 3:**
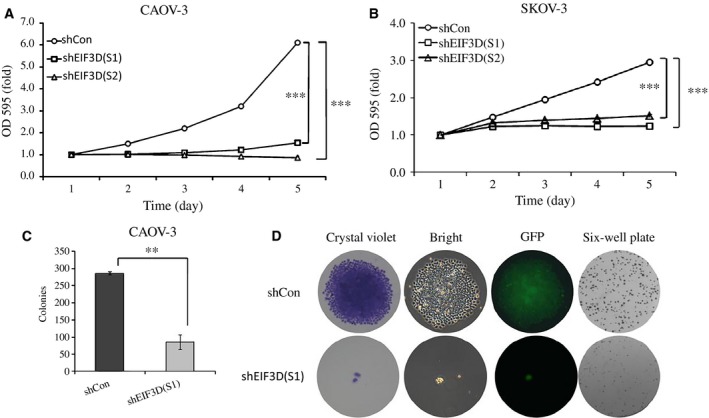
Effect of EIF3D down‐regulated on cell proliferation and colony formation**.** Proliferation of CAOV‐3 (A) and SKOV‐3 (B) cells was observed by MTT assay after transfected with Lv‐shEIF3D for 1, 2, 3, 4 and 5 days. Clonogenesis ability of CAOV‐3 cells was observed by colony formation assay after transfected with Lv‐shEIF3D. Average number of colonies of each group (C) and representative photographs of single colony and colonies in six‐well plates (D) was shown. **P < 0.001, compared with shCon. ***P < 0.01, compared with shCon.

Down‐regulation of EIF3D produced grotesque and less‐adherent cells than the cells in the shCon group (Fig. 2A,D). A MTT assay and colony formation assay were performed to determine the function of EIF3D in cell proliferation. After transfection with Lv‐shEIF3D for 5 days, the growth of CAOV‐3 cells (Fig. 3A) and SKOV‐3 cells (Fig. 3B) was significantly reduced (*P* < 0.001). Furthermore, a clonogenic assay of the CAOV‐3 cells was performed after infection, and fewer colonies were observed in the shEIF3D group than in the shCon group (Fig. 3C, 85 ± 22 vs. 286 ± 5, *P* = 0.00257) and the size and number of cells in a single colony were reduced (Fig. 3D).

### Down‐regulation of EIF3D induces S phase reduction and G2/M phase arrest in CAOV‐3 cells

**Figure 4:**
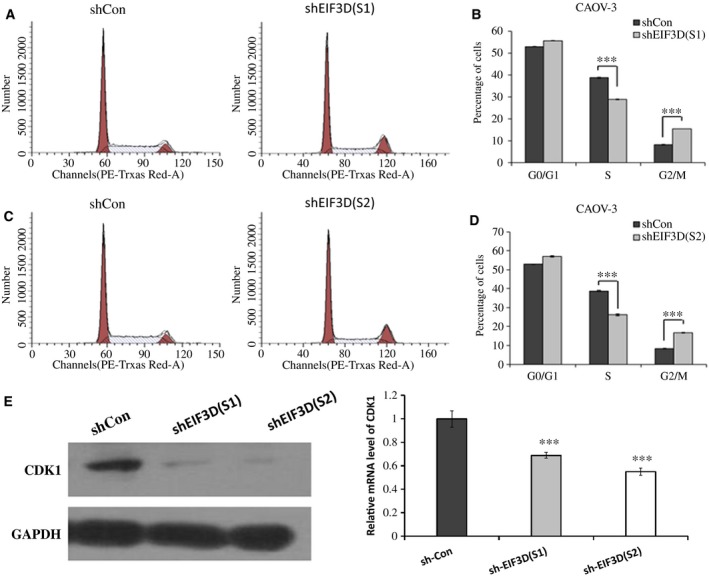
Down‐regulation of EIF3D induces S phase reduction and G2/M phase arrest. Cell cycle distribution of CAOV‐3 cells was analysed by Flow cytometry (A, C). The proportion of each phase in cell cycle was shown as histogram (B, D). mRNA and protein level of CDK1 was decreased in shEIF3D CAOV‐3 cells (E). ***P < 0.001, compared with shCon.

Uncontrolled cell cycles are related to malignancy transformation and tumourigenesis, and inhibition of cell cycle progression is a promising method of inhibiting carcinogenesis. To investigate changes in the progression of the cell cycle in EIF3D‐down‐regulated CAOV‐3 cells, we analysed the specimens via flow cytometry (Fig. 4A, C). The EIF3D‐down‐regulated cells were inhibited at the G2/M phase (Fig. 4B, D, *P* < 0.001), and the proportion of cells in the S phase was significantly decreased (Fig. 4B, D, *P* < 0.001). Because these results might be explained via the modulation of cell cycle‐related proteins, we detected the expression of corresponding genes and proteins and found that the levels of CDK1 mRNA and proteins were down‐regulated after silencing EIF3D (Fig. 4E).

## Discussion

Gene expression is a sophisticated process that is mainly regulated at the transcription and translation levels in eukaryotes. Deregulated translation may lead to alterations in gene expression, and an abnormal level of proteins can enhance the proliferative activity of cells and result in immortalization 7. The initial period of mRNA translation plays an important role in regulating protein synthesis and controlling cell proliferation and differentiation.

Eukaryotic translation initiation factor dysregulation is commonly observed in gynaecologic malignancies. For example, increased EIF5A2 expression is an independent molecular marker for reduced survival in patients with ovarian carcinoma 22 and EIF4E is a useful marker for malignant transformation of cervical squamous cells 23. As the largest and most complex initiation factor, EIF3 acts as a scaffold for the recruitment of mRNA to the 40S ribosomal subunit, and it stabilizes the ternary complex by binding to the 40S subunit. An attractive hypothesis holds that altered levels of EIF3 subunits may stimulate the signal transduction of over proliferation and malignant transformation, which has been observed in a variety of cancers 24, 25, 26.

Eukaryotic translation initiation factor 3 subunit D is a non‐core subunit of EIF3 that has received minimal attention to date. EIF3D is involved in the stabilization of EIF3 subunits 16 and the inhibition of HIV replication 17. A prospective, high‐throughput transcriptional profiling study was performed to investigate the relationship between EIF3D and different cancers, and the results indicated that EIF3D is up‐regulated in gastric cancer patients resistant to cisplatin and fluorouracil combination chemotherapy 27. In another large‐scale screening, EIF3D knockdown was shown to be involved in the anti‐apoptotic functions in mesothelioma 28. However, these studies were narrow in scope and lacked validation. Further studies were recently performed, and the lentivirus‐mediated knockdown of EIF3D was found to suppress cell proliferation in human melanoma 18 and colon tumours 20, which indicated a possible role of EIF3D as an oncogene.

In this study, EIF3D was overexpressed in ovarian cancer clinical tissues compared with benign ovarian cystadenoma and borderline cystadenoma. Furthermore, EIF3D expression was associated with the FIGO stage and pathological differentiation stage, which suggested that EIF3D expression correlates with the occurrence and development of ovarian cancer. *In vitro* studies have indicated that cell proliferation and colony formation is suppressed after lentivirus‐mediated knockdown of EIF3D. Similar to the results of previous studies 18, 20, our investigation found that cells were arrested at the G2/M phase of the cell cycle after EIF3D knockdown. The qPCR and western blot results further indicated that EIF3D knockdown reduces CDK1 gene and protein expression. CDK1, which is also known as CDC2, belongs to the Ser/Thr protein kinase family and participates in the formation of M phase promoting factor, which is essential for G1/S and G2/M phase transitions in the eukaryotic cell cycle 29, 30. Phosphorylation and dephosphorylation of CDK1 play important regulatory roles in the cell cycle 31, and CDK1 activity is often related to cancer 32, 33. Thus, we conclude that deregulation of EIF3D is associated with the occurrence and development of ovarian cancer, and our results provide the first evidence that lentivirus‐mediated knockdown of EIF3D inhibits the proliferation of ovarian tumour cell lines by inducing G2/M cell cycle arrest. Considerable advantages will be obtained from further studies of this potential biomarker and therapeutic target of ovarian cancer.

## Author contributions

YL, RZ and PZ conceived and designed the project, acquired the data, analysed and interpreted the data, and wrote the paper together.
